# Anaerobic metabolic responses of sludge and mine wastewater-derived consortia to winery wastewater-related compounds

**DOI:** 10.1007/s11274-026-05195-3

**Published:** 2026-08-18

**Authors:** Ana Baía, Alonso I. Arroyo-Escoto, Nuno Ramos, César Afonso, Gaetano R. Pesce, Maria C. Fernandes, Annabel Fernandes

**Affiliations:** 1 Fiber Materials and Environmental Technologies (FibEnTech-UBI), Universidade da Beira Interior, Covilhã, Portugal; 2 Department of Chemistry, Universidade da Beira Interior, Covilhã, Portugal; 3 Alentejo Biotechnology Center for Agriculture and Agro-Food (CEBAL), Polytechnic Institute of Beja (IPBeja), Beja, Portugal; 4 MED – Mediterranean Institute for Agriculture, Environment and Development & CHANGE – Global Change and Sustainability Institute, CEBAL- Alentejo Biotechnology Center for Agriculture and Agro- Food, Beja, Portugal; 5 Institute for Advanced Studies and Research (IIFA), MED – Mediterranean Institute for Agriculture, Environment and Development & CHANGE– Global Change and Sustainability Institute, University of Évora, Évora, Portugal; 6 Department of Agronomy, Natural Resources, Animals and Environment—DAFNAE, University of Padua, Agripolis Campus, Food, Legnaro, PD Italy

**Keywords:** Agro-industrial wastewater, Anaerobic metabolism, Full-length 16S rRNA sequencing, Fumarate respiration, Mixed microbial communities, Phenolic stress

## Abstract

Winery wastewater contains readily biodegradable organic matter, but some of its components can inhibit anaerobic microbial activity. This study compares two enriched microbial consortia, derived from anaerobic digester sludge (ADS) and mining-process wastewater (MPW), exposed to alcohols, carboxylic acids, and phenolics. Assays were conducted in acetate–fumarate-based media containing low or high concentrations of each compound class and a combined condition containing the compounds at their highest levels. Growth, fumarate consumption, succinate production, acetate turnover, and compound consumption were monitored. Full-length 16 S rRNA gene sequencing showed that ADS was dominated by *Proteus* (56.9%) and *Escherichia* (28.6%). MPW showed a more even profile dominated by *Proteus* (35.8%), *Wolinella* (32.9%), and *Escherichia* (23.0%). Under control conditions, MPW showed higher growth (0.284 vs. 0.181 h^−1^) and succinate production rates (0.171 vs. 0.106 g L^−1^ h^−1^) than ADS. Alcohols caused little change in fumarate consumption, but high carboxylic acid concentrations reduced fumarate consumption rates to 0.115 and 0.162 g L^−1^ h^−1^ in ADS and MPW, respectively. Phenolics reduced the ADS succinate production rate to 0.059 g L^−1^ h^−1^, while MPW remained near its control value (0.169 vs. 0.171 g L^−1^ h^−1^). Under combined exposure, ADS maintained fumarate consumption and increased succinate production from 0.106 to 0.157 g L^−1^ h^−1^, but MPW decreased from 0.125 to 0.092 g L^−1^ h^−1^. Both bacterial composition and the chemical conditions of exposure influenced anaerobic resilience. The combined use of taxonomic and metabolic indicators may therefore improve the selection of consortia for the biodegradation and valorization of complex agro-industrial wastewaters, including their integration into bioelectrochemical platforms.

**Supplementary Information:**

The online version contains supplementary material available at https://doi.org/10.1007/s11274-026-05195-3.

## Introduction

The highly biodegradable organic load of winery wastewater makes it a suitable substrate for biological valorization processes, in which microbial communities can convert organic matter into biomass, metabolites, or recoverable energy carriers (Nayak et al. 2024). This is particularly relevant to anaerobic biological and bioelectrochemical processes, including microbial electrolysis cells, which can couple organic matter oxidation with energy recovery (e.g., hydrogen production) while relying on electroactive microbial communities (Call and Logan 2008; Jafary et al. 2019; Priyadarshini et al. 2022). In such systems, the development of syntrophic and electroactive communities can influence electron recovery, organic matter degradation, and overall process performance (Toczyłowska-Mamińska et al. 2020; Wang et al. 2022). However, the valorization of winery wastewater is challenged by the chemical complexity of the effluent, which combines readily biodegradable substrates with compounds that can inhibit microbial activity (Baía et al. 2022). These compounds include alcohols, organic acids, and phenolics, whose concentrations vary with grape variety, wine type, production technology, cleaning operations, and the winemaking season (Liu et al. 2021; Ngwenya et al. 2022; Melchiors and Freire 2023). Efficient biological or bioelectrochemical valorization depends on microbial communities that remain active in the presence of such compounds and are also able to transform them.

Anaerobic mixed microbial communities are especially important because they combine complementary metabolic functions that are rarely found in a single strain, a key advantage for degrading complex organic compounds (Cao et al. 2022). Through fermentative and syntrophic interactions, such communities can transform complex organic substrates into intermediates such as volatile fatty acids, succinate, acetate, hydrogen, and carbon dioxide (Pan et al. 2021; Nikitina et al. 2023; Parshina et al. 2023; Lee et al. 2024). The accumulation or consumption of the intermediates also provides information on anaerobic carbon flow and redox balance. However, the structure and activity of anaerobic communities are strongly influenced by substrate composition and by the presence of inhibitory compounds, which can redirect metabolic routes, modify intermediate accumulation, and affect overall biodegradation performance (Logan et al. 2023; Gaspar et al. 2025). In this context, fumarate consumption, succinate formation, and acetate turnover provide useful functional indicators of anaerobic metabolic activity. Fumarate can act as an electron acceptor under anaerobic conditions, and its reduction to succinate reflects metabolic responses associated with cellular redox balance that may be altered by inhibitory compounds (Schubert and Unden 2022, 2023).

The organic compounds present in winery wastewater can play different roles in anaerobic biodegradation, acting as substrates, metabolic intermediates, or inhibitory agents depending on their chemical nature and concentration. Alcohols such as ethanol and glycerol contribute to the biodegradable organic load and may serve as electron donors or fermentation substrates (Liu et al. 2021; Parshina et al. 2023). Carboxylic acids, including acetic, lactic, tartaric, and succinic acids, participate in anaerobic metabolic networks but, at elevated concentrations, may disturb intracellular pH, redox balance, or substrate uptake (Pan et al. 2021; Schubert and Unden 2022; Gaspar et al. 2025). Phenolic compounds are generally more recalcitrant and may inhibit microbial activity by affecting enzymes, membranes, and electron transfer processes. Their anaerobic biodegradation often requires specialized pathways for aromatic ring activation and cleavage (Schink et al. 2000; Levén et al. 2012; Tomei et al. 2021). Because the compound classes occur together, growth alone may not describe the microbial response. Changes in carbon conversion and substrate use must also be considered.

The response of microbial communities to mixed substrate–inhibitor pressure is shaped by their ecological origin and previous selective history. Communities enriched in anaerobic digester sludge are typically associated with organic matter degradation, fermentation, syntrophic interactions, and the transformation of volatile fatty acids. By contrast, microbial communities from mine wastewater may be shaped by acidic, metal-rich, and chemically stressful environments, where redox transformations involving metals, sulfur compounds, and mineral surfaces contribute to microbial selection (Candeias et al. 2014; Pattanaik et al. 2020; Shi et al. 2016). Comparing the two inocula allow to examine whether their different environmental origins are associated with different patterns of tolerance and anaerobic carbon conversion. This distinction is important because two communities may exhibit similar substrate consumption yet rely on different metabolic strategies, resulting in distinct patterns of intermediate production, accumulation, or consumption.

In the present study, two enriched microbial consortia derived from anaerobic digester sludge (ADS) and mining process wastewater (MPW) were compared using defined nutrient broth acetate fumarate (NBAF)-based model media containing alcohols, carboxylic acids, and phenolic compounds associated with winery wastewater. Each compound class was evaluated separately at low and high concentrations, and all classes were subsequently combined at their highest tested concentrations. The bacterial composition and diversity of the enriched consortia were characterized by full-length 16 S rRNA gene sequencing. The research objective was to evaluate whether two enriched consortia differing in inoculum source and resulting bacterial composition exhibited distinct tolerance and anaerobic metabolic responses to compounds associated with winery wastewater, as assessed by growth, fumarate consumption, succinic acid production, acetate turnover, and compound consumption. By integrating taxonomic and functional data, the study sought to identify response patterns relevant to the selection of microbial consortia for the biodegradation and sustainable valorization of complex agro-industrial wastewaters, including their future integration into bioelectrochemical platforms.

## Methods

### Microbial consortia and enrichment procedure

The MPW consortium was obtained from mine wastewater collected at the Panasqueira mine area, Barroca Grande, Covilhã, Portugal. The ADS consortium was obtained from sludge collected from an anaerobic digestion system located in Baleizão, Beja, Portugal. After collection, the samples were stored at (4 ± 1) °C until use to preserve microbial activity.

For consortium enrichment, 5 mL of each original sample was inoculated into 70 mL of NBAF medium. The medium was prepared according to Huerta-Miranda et al. (2019), with 30 mM sodium acetate and 40 mM sodium fumarate, and was supplemented with 0.5 g L^−1^ yeast extract and 0.12 g L^−1^ cysteine. This medium was selected because it supports the enrichment of anaerobic microbial communities under conditions suitable for bioelectrochemical applications (O’Brien and Malvankar 2016).

Anaerobic conditions were established by sparging the medium with an N_2_/CO_2_ gas mixture (80:20, v/v) for 15 min. The medium was then sterilized by autoclaving at 121 °C for 20 min. The inoculated cultures were incubated statically at 36 °C in a thermostatic cabinet. The enrichment process was maintained for 3 months by transferring 5 mL of culture into fresh NBAF medium at 5-day intervals. Repeated transfers over three months enriched populations that remained active under anaerobic conditions.

The original mine wastewater and anaerobic sludge samples were used solely as inoculum sources for consortium enrichment. All subsequent fermentation assays were conducted with the enriched cultures in NBAF-based synthetic media.

### Bacterial community characterization

The enriched ADS and MPW consortia were submitted to an external sequencing service for bacterial taxonomic characterization based on the full-length 16 S rRNA gene (STAB VIDA Lda., Portugal). Total DNA was extracted using the Maxwell^®^ RSC PureFood GMO and Authentication Kit according to the manufacturer’s instructions. Full-length 16 S rRNA gene amplification was performed using primers 27 F (5′-AGAGTTTGATCMTGGCTCAG-3′) and 1492R (5′-CGGTTACCTTGTTACGACTT-3′), covering the V1–V9 hypervariable regions. Sequencing libraries were prepared using the 16 S Barcoding Kit V14 (SQK-16S114.24; Oxford Nanopore Technologies) and sequenced on a MinION Mk1D platform using a Flongle Flow Cell. Basecalling was performed in Super Accuracy mode using model version 5.2.0 at 400 bases s^− 1^. Taxonomic classification and relative abundance estimation were performed using the wf-emu workflow provided by Oxford Nanopore Technologies (Curry et al. 2022). The workflow used a combined database composed of rrnDB version 5.10 and NCBI 16 S RefSeq. Reads were aligned using minimap2, retaining up to 50 alignments per read, and multi-mapped reads were subsequently resolved using an expectation-maximization algorithm (Li 2018). Taxa were retained only when their relative abundance was ≥ 0.01%, thereby reducing the contribution of low-abundance false-positive assignments and sequencing-associated noise. Relative abundances at the phylum, family, and genus levels were calculated by summing the estimated read counts assigned to the corresponding taxonomic group. Genus-level diversity was described using observed genus richness, the Shannon diversity index, the Simpson diversity index expressed as 1 − D, and Pielou’s evenness index. Because one sequencing library was analyzed for each enriched consortium, taxonomic composition and diversity were compared descriptively, without inferential statistical testing. No individual strains were isolated. Because the analysis was based on 16 S rRNA gene sequencing for bacterial taxonomic characterization, fungal communities were not assessed.

### Fermentation experiments

Fermentation assays were performed using the NBAF basal medium, prepared as described in Sect.  2.1. To simulate, under controlled conditions, the main classes of compounds associated with winery wastewater, the medium was supplemented with mixtures of alcohols, carboxylic acids, or phenolic compounds. Each compound class was tested independently at two final concentration levels, designated low and high.

The alcohol mixture contained ethanol, glycerol, and methanol. The carboxylic acid mixture contained L(+)-tartaric, lactic, acetic, and succinic acids. The phenolic mixture contained tyrosol and catechol. A combined condition was also prepared by adding all tested compounds simultaneously at their highest concentrations. Control assays consisted of NBAF basal medium without the addition of the test compounds. The composition and initial physicochemical characteristics of the control and supplemented experimental media are presented in Table 1.

**Table 1:** Composition and physicochemical characteristics of the synthetic media used in the fermentation assays

	Control	Alc-L	Alc-H	Acid-L	Acid-H	Ph-L	Ph-H	Comb-H
Added compound concentration (g L^−1^)								
Ethanol	−	2.5	5.0	−	−	−	−	5.0
Glycerol	−	0.16	0.32	−	−	−	−	0.32
Methanol	−	0.0075	0.015	−	−	−	−	0.015
L(+)-tartaric acid	−	−	−	0.26	0.53	−	−	0.53
Lactic acid	−	−	−	0.18	0.35	−	−	0.35
Acetic acid	−	−	−	0.33	0.66	−	−	0.66
Succinic acid	−	−	−	0.04	0.08	−	−	0.08
Tyrosol	−	−	−	−	−	0.36	0.72	0.72
Catechol	−	−	−	−	−	0.36	0.72	0.72
Physicochemical characteristics								
Chemical oxygen demand (g O_2_ L^−1^)	6.23±0.02	11.6±0.1	17.8±0.1	6.92±0.03	7.65±0.08	7.8±0.3	9.26±0.04	22.8±0.1
Dissolved organic carbon (g C L^−1^)	2.46±0.04	3.77±0.02	5.20±0.02	2.75±0.04	3.08±0.03	2.95±0.08	3.40±0.02	6.56±0.06
Dissolved inorganic carbon (g C L^−1^)	0.31±0.03	0.32±0.04	0.34±0.03	0.19±0.05	0.07±0.02	0.31±0.02	0.29±0.03	0.10±0.04
Total dissolved nitrogen (mg N L^−1^)	131±4	134±2	135±5	140±3	139±7	142±6	130±2	128±4
Total phenolic content (mg tannic acid equivalents L^−1^)	−	−	−	−	−	(60±4)⋅10	(131±5)⋅10	(133±7)⋅10
Orthophosphate (mg PO_4_^3−^ L^−1^)	(42±2)⋅10	(40±3)⋅10	(42±1)⋅10	(44±4)⋅10	(40±3)⋅10	(41±2)⋅10	(43±1)⋅10	(41±3)⋅10
pH	7.04±0.08	7.10±0.06	7.11±0.04	6.60±0.04	6.02±0.09	7.06±0.05	7.04±0.06	6.80±0.07
Electrical conductivity (mS cm^−1^)	14.5±0.3	14.2±0.5	14.3±0.1	14.5±0.1	14.2±0.3	14.5±0.2	14.6±0.2	14.0±0.4

All conditions contained NBAF basal medium supplemented with 0.5 g L^−1^ yeast extract and 0.12 g L^−1^ cysteine. Alc-L and Alc-H, low- and high-concentration alcohol media; Acid-L and Acid-H, low- and high-concentration carboxylic-acid media; Ph-L and Ph-H, low- and high-concentration phenolic media; Comb-H, combined medium containing all compounds at their highest tested concentrations; −, not added or not detected

The concentrations of the tested compounds were selected based on values reported for winery wastewater and were intended to represent both environmentally pertinent levels and intensified stress conditions. Ethanol is commonly present in winery effluents and may occur at concentrations between 2.5 and 8 g L^−1^, contributing substantially to the organic load (Colin et al. 2005). Organic acids, including tartaric, lactic, and succinic, are frequently detected at concentrations ranging from several hundred mg L^−1^ to more than 1 g L^−1^, depending on the winemaking process and seasonal conditions (Welz et al. 2016). Phenolic compounds, including tyrosol and catechol derivatives, are generally present at lower concentrations, with total phenolic contents commonly reported in the range of 10–100 mg L^−1^, although higher values may occur depending on grape processing and wastewater composition (Zacharof 2017; Cañadas et al. 2021). Thus, the concentrations used in this study were selected to evaluate the ability of the consortia to maintain anaerobic activity under increased substrate–inhibitor pressure in addition to representing compounds related to winery wastewater. The combined medium was also tested to examine the response to all compound classes present simultaneously.

The assays were carried out in 150 mL anaerobic culture flasks containing 70 mL of NBAF basal medium. Each flask was supplemented with 1 mL of the appropriate concentrated stock solution and inoculated with the corresponding enriched consortium at a volume adjusted to obtain an initial optical density at 600 nm (OD_600_) of approximately 0.4. The final working volume was approximately 80 mL. Each condition was evaluated in two independently prepared fermentation flasks, which were incubated statically at 36 °C.

During the fermentation assays, liquid samples were collected periodically for OD_600_ measurement and high-performance liquid chromatography (HPLC) analysis. Immediately before sampling, the fermentation flasks were gently mixed to homogenize the culture. Samples of 2 mL were withdrawn aseptically using sterile syringes in a laminar flow chamber. During the first 12 h of incubation, samples were collected hourly. Thereafter, the sampling interval was progressively increased, typically to 2 h, according to the evolution of the fermentation profiles. OD_600_ was measured immediately after sampling. The remaining sample was then centrifuged at 5000 rpm for 5 min to remove microbial cells, and the supernatant was diluted as required before HPLC analysis.

### Analytical methods and calculations

Before inoculation, the control and inhibitor-containing synthetic media were characterized in terms of chemical oxygen demand (COD), dissolved organic carbon (DOC), dissolved inorganic carbon (DIC), total dissolved nitrogen (TDN), total phenolic content, orthophosphate, pH, and electrical conductivity. COD determination followed the closed reflux titrimetric method, according to the standard procedures (Eaton et al. 2005). DOC, DIC, and TDN were measured using a Shimadzu TOC-VCPH analyzer combined with a TNM-1 unit. Before the analysis, samples were filtered through 1.2-µm glass microfiber membranes. Total phenolic content, expressed as tannic acid equivalents, was determined following the procedure described by Makkar et al. (1993). Orthophosphate was determined by ion chromatography using a Shine CIC-D260 system equipped with an SH-GP-2 Shine (4.0 × 50 mm) anion guard column and an SH-AP-1 Shine (4.0 × 250 mm) anion column. The mobile phase was a solution of 17.0 mM sodium hydroxide, with a flow rate of 0.7 mL min^− 1^. The column temperature was 35 °C. Before analysis, samples were filtered through 0.45 μm polypropylene membrane filters. pH and electrical conductivity were measured using a HANNA pH meter HI 931,400 and a Mettler Toledo conductivity meter SevenEasy S30K, respectively. Measurements were performed in three analytical replicates, and the results are expressed as mean ± standard deviation.

Microbial growth was monitored by measuring OD_600_ using a Thermo Scientific Evolution 201 UV–Visible spectrophotometer. The specific growth rate, µ, expressed in h^−1^, was calculated from the linear region of the exponential growth phase using Eq. (1), where OD_600(0)_ and OD_600(t)_ represent the optical density at time 0 and at time t (h), respectively.

1 $$\:{ln}\left(\frac{{{O}{D}}_{600\left({t}\right)}}{{{O}{D}}_{600\left(0\right)}}\right)={\upmu\:}\:{t}$$

The concentrations of the tested alcohols, carboxylic acids, and phenolic compounds, as well as fumarate and the main fermentation products, were quantified by HPLC using a Shimadzu system equipped with a refractive index detector, RID-20 A. Separation was performed using a Bio-Rad Aminex HPX-87 H cation-exchange column operated under isocratic conditions. Sulfuric acid 5 mM was used as the mobile phase at a flow rate of 0.6 mL min^−1^, and the column oven was maintained at 50 °C. Quantification was carried out using calibration curves prepared for each compound.

The consumption rate of each compound (Q_CX_), expressed in g L^−1^ h^−1^, was calculated using Eq. (2), where Cx_0_ and Cx_t_ (g L^−1^) are the concentrations of compound X at time 0 and at time t (h), respectively.

For fermentation products, production rates were determined using the same equation, with the numerator terms reversed. Rate calculations were based on the time required to reach the maximum or minimum concentration of each compound, with t denoting the time interval.

2 $$\:{{Q}}_{{C}{X}}=\frac{{{C}{x}}_{0}-{{C}{x}}_{{t}}}{{t}}$$

### Statistical analysis

Growth, consumption, and production rates were statistically analyzed using analysis of variance (ANOVA). The analyses were performed using the individual replicate values. For the assays in which each inhibitor class was tested independently, two-way ANOVA was performed with consortium and inhibitor concentration as fixed factors, including the consortium × concentration interaction. Inhibitor concentration comprised three levels: control without added inhibitors, low concentration, and high concentration. For the combined inhibitor assays, two-way ANOVA was performed with consortium and inhibitor condition as fixed factors, including the consortium × condition interaction. The inhibitor condition comprised two levels: control without added inhibitors and combined inhibitors at their highest tested concentrations. Given that each consortium–condition combination was evaluated in duplicate, the ANOVA results were interpreted as exploratory indicators of main and interaction effects rather than as definitive evidence of differences between individual treatment means.

## Results

### Baseline behavior of the consortia in NBAF medium

**Fig. 1:**
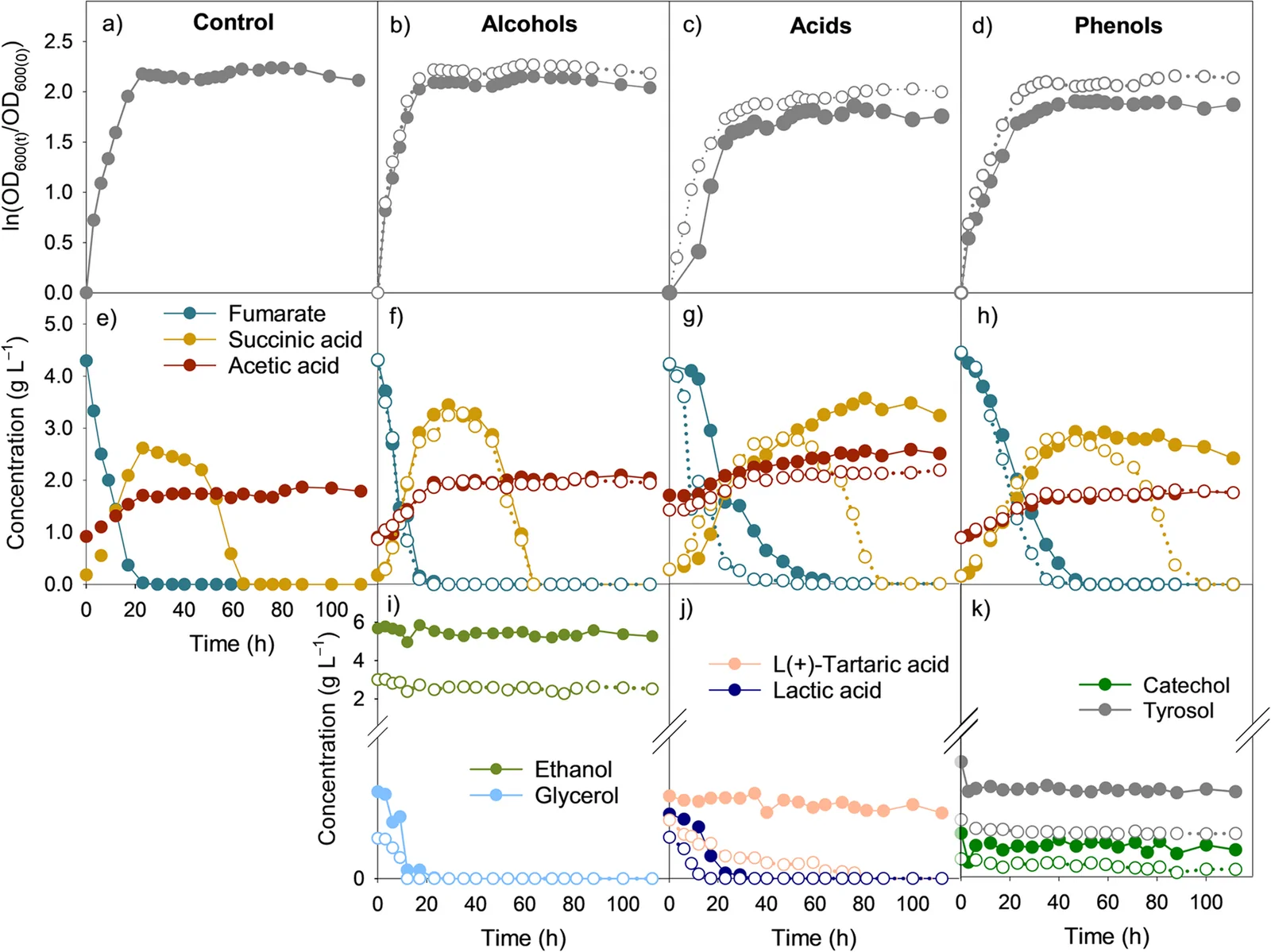
Growth curves and fermentation profiles of the ADS consortium in NBAF medium under control conditions, without inhibitors (**a**, **e**), and in the presence of alcohols (**b**, **f**, **i**), carboxylic acids (**c**, **g**, **j**), and phenolic compounds (**d**, **h**, **k**). In inhibitor assays, full symbols represent high concentration and open symbols represent low concentration. Error bars were omitted for clarity because replicate values generally differed by less than 10% for the monitored parameters, without affecting the interpretation of the observed trends

**Fig. 2:**
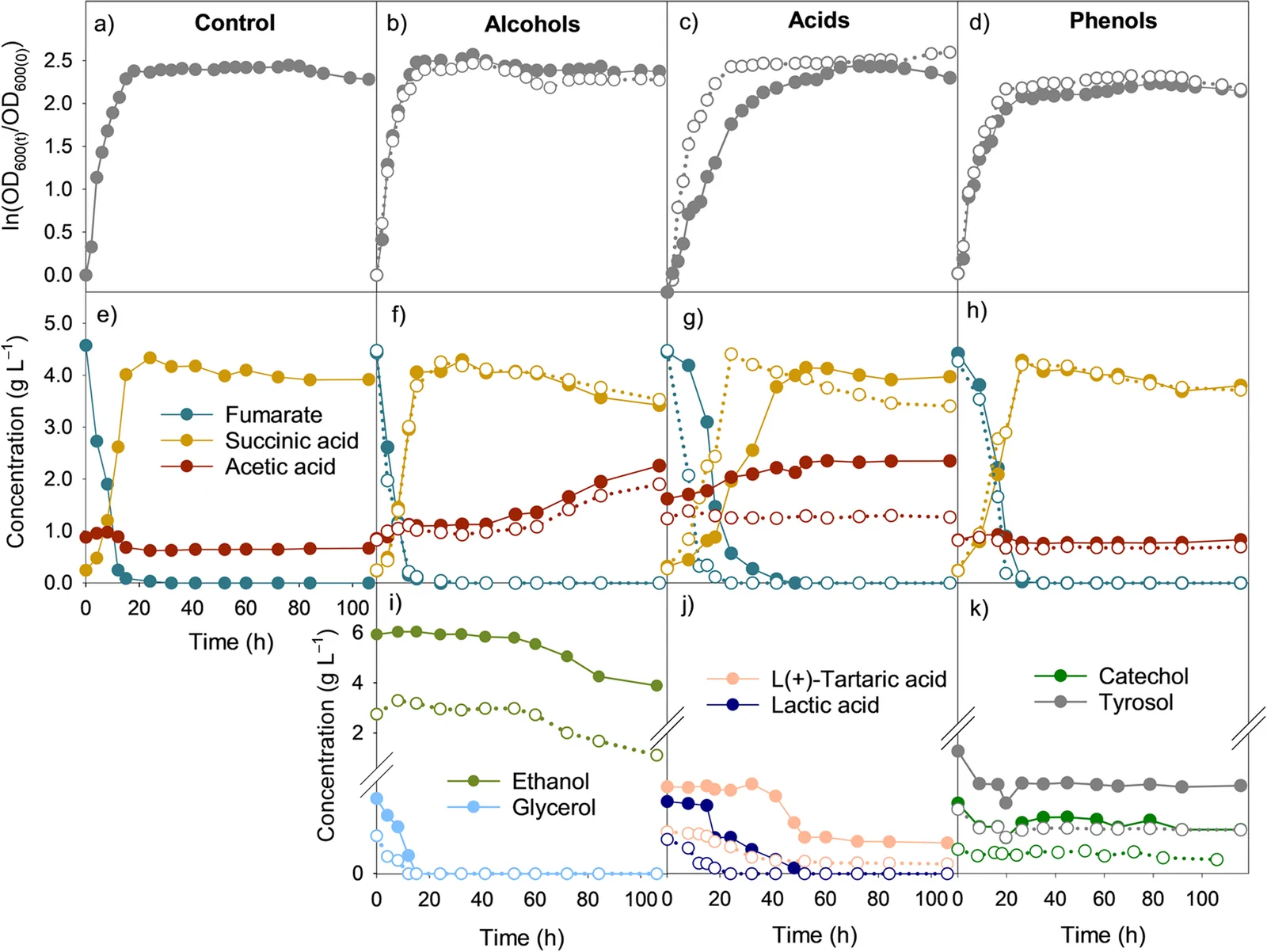
Growth curves and fermentation profiles of the MPW consortium in NBAF medium under control conditions, without inhibitors (**a**, **e**), and in the presence of alcohols (**b**, **f**, **i**), carboxylic acids (**c**, **g**, **j**), and phenolic compounds (**d**, **h**, **k**). In inhibitor assays, full symbols represent high concentration and open symbols represent low concentration. Error bars were omitted for clarity because replicate values generally differed by less than 10% for the monitored parameters, without affecting the interpretation of the observed trends

Control assays without added inhibitors were performed in NBAF medium to establish the baseline growth and fermentation behavior of both consortia. Figures 1a and e and 2a and e present the growth and fermentation profiles of the ADS and MPW consortia, respectively. Under control conditions, both consortia showed rapid growth, with an exponential phase during the first 10–12 h followed by a stationary phase. However, MPW showed a higher control growth rate than ADS, 0.284 vs. 0.181 h^− 1^. The overall effect of the consortium on growth rate was highly significant in each of the single-class inhibitor analyses (*p* < 0.001; Tables 2, 3 and 4).

**Table 2:** Effects of alcohol concentration on the growth and metabolic rates of microbial consortia derived from ADS and MPW. The reported p-values correspond to the effects of consortium, alcohol concentration, and the consortium × concentration interaction, as determined by two-way analysis of variance. Negative acetic acid production rates indicate net acetate consumption

Variable		Consortium	Control	Alcohol compound concentration		p-value	
				Low	High		
Growth rate (h^-^^1^)		ADS	0.181	0.217	0.190	Consortium Inhibitor Consortium ⨯ Inhibitor	<0.001 0.094 0.168
		MPW	0.284	0.302	0.322		
Consumption rate (g L^-^^1^ h^-^^1^)	Fumarate	ADS	0.186	0.188	0.185	Consortium Inhibitor Consortium ⨯ Inhibitor	0.815 0.153 0.073
		MPW	0.189	0.185	0.185		
	Ethanol	ADS	0.000	0.004	0.005	Consortium Inhibitor Consortium ⨯ Inhibitor	<0.001 <0.001 0.005
		MPW	0.000	0.015	0.019		
	Glycerol	ADS	0.000	0.012	0.022	Consortium Inhibitor Consortium ⨯ Inhibitor	<0.001 <0.001 0.003
		MPW	0.000	0.014	0.027		
Production rate (g L^-^^1^ h^-^^1^)	Acetic acid	ADS	0.028	0.033	0.039	Consortium Inhibitor Consortium ⨯ Inhibitor	0.001 0.128 0.646
		MPW	- 0.011	0.006	0.011		
	Succinic acid	ADS	0.106	0.117	0.134	Consortium Inhibitor Consortium ⨯ Inhibitor	0.023 0.216 0.024
		MPW	0.171	0.123	0.133

**Table 3:** Effects of carboxylic acid concentration on the growth and metabolic rates of microbial consortia derived from ADS and MPW. The reported p-values correspond to the effects of consortium, carboxylic acid concentration, and the consortium × concentration interaction, as determined by two-way analysis of variance. Negative acetic acid production rates indicate net acetate consumption

Variable		Consortium		Carboxylic acid concentration		p-value	
			Control	Low	High		
Growth rate (h^-^^1^)		ADS	0.181	0.113	0.106	Consortium Inhibitor Consortium ⨯ Inhibitor	<0.001 <0.001 0.002
		MPW	0.284	0.219	0.085		
Consumption rate (g L^-^^1^ h^-^^1^)	Fumarate	ADS	0.186	0.167	0.115	Consortium Inhibitor Consortium ⨯ Inhibitor	0.003 <0.001 0.029
		MPW	0.189	0.186	0.162		
	Lactic acid	ADS	0.000	0.022	0.018	Consortium Inhibitor Consortium ⨯ Inhibitor	<0.001 <0.001 <0.001
		MPW	0.000	0.010	0.010		
	Tartaric acid	ADS	0.000	0.006	0.001	Consortium Inhibitor Consortium ⨯ Inhibitor	<0.001 <0.001 <0.001
		MPW	0.000	0.005	0.007		
Production rate (g L^-^^1^ h^-^^1^)	Acetic acid	ADS	0.028	0.010	0.011	Consortium Inhibitor Consortium ⨯ Inhibitor	<0.001 0.165 0.002
		MPW	- 0.011	0.001	0.013		
	Succinic acid	ADS	0.106	0.061	0.041	Consortium Inhibitor Consortium ⨯ Inhibitor	<0.001 <0.001 <0.001
		MPW	0.171	0.172	0.074

**Table 4:** Effects of phenolic compound concentration on the growth and metabolic rates of microbial consortia derived from ADS and MPW. The reported p-values correspond to the effects of consortium, phenolic compound concentration, and the consortium × concentration interaction, as determined by two-way analysis of variance. Negative acetic acid production rates indicate net acetate consumption

Variable		Consortium		Phenolic compound concentration		p-value	
			Control	Low	High		
Growth rate (h^-^^1^)		ADS	0.181	0.081	0.063	Consortium Inhibitor Consortium ⨯ Inhibitor	<0.001 0.001 0.425
		MPW	0.284	0.216	0.204		
Consumption rate (g L^-^^1^ h^-^^1^)	Fumarate	ADS	0.186	0.139	0.105	Consortium Inhibitor Consortium ⨯ Inhibitor	<0.001 <0.001 <0.001
		MPW	0.189	0.173	0.183		
	Catechol	ADS	0.000	0.002	0.080	Consortium Inhibitor Consortium ⨯ Inhibitor	0.008 <0.001 0.002
		MPW	0.000	0.007	0.024		
	Tyrosol	ADS	0.000	0.153	0.081	Consortium Inhibitor Consortium ⨯ Inhibitor	<0.001 <0.001 <0.001
		MPW	0.000	0.019	0.033		
Production rate (g L^-^^1^ h^-^^1^)	Acetic acid	ADS	0.028	0.017	0.017	Consortium Inhibitor Consortium ⨯ Inhibitor	<0.001 0.578 0.043
		MPW	- 0.011	- 0.007	- 0.002		
	Succinic acid	ADS	0.106	0.055	0.059	Consortium Inhibitor Consortium ⨯ Inhibitor	<0.001 <0.001 <0.001
		MPW	0.171	0.165	0.169		

Fumarate was rapidly consumed by both consortia. Complete or near-complete fumarate depletion occurred within approximately 17 h for MPW and 23 h for ADS. Fumarate consumption rates were similar in both consortia, 0.189 and 0.186 g L^−1^ h^−1^ for MPW and ADS, respectively (Tables 2, 3 and 4). However, the fermentation product profiles differed between consortia. MPW showed a higher succinic acid production rate than ADS, 0.171 vs. 0.106 g L^−1^ h^−1^, respectively (Table 2), and succinic acid remained relatively stable after fumarate conversion. In contrast, ADS subsequently consumed succinic acid, which was depleted after approximately 64 h, while acetic acid increased during the first 24 h and then remained stable (Fig. 1e).

## Response to single inhibitor classes

### Alcohols

The effects of alcohols on growth, fumarate consumption, ethanol and glycerol consumption, and succinic and acetic acid production are summarized in Table 2, while the individual duplicate values underlying the reported means are provided in Supplementary Table S1. The corresponding growth and fermentation profiles are shown in Fig. 1b, f,i for ADS and Fig. 2b, f,i for MPW. Methanol was included in the alcohol mixture, but because of its low concentration, it could not be reliably monitored by HPLC.

Alcohol exposure did not markedly alter the overall growth pattern of either consortium. The main effect of the consortium was highly significant for growth rate (*p* < 0.001), although the effects of alcohol concentration and the consortium × alcohol interaction were not significant (Table 2). MPW maintained higher growth rates than ADS under all conditions, increasing from 0.284 h^−1^ in the control to 0.322 h^−1^ at high alcohol concentration. ADS showed lower growth rates, ranging from 0.181 to 0.217 h^− 1^, and no significant overall effect of alcohol concentration on growth rate was detected.

Fumarate consumption was highly stable in the presence of alcohols. No significant effects were detected for consortium, alcohol concentration, or their interaction, and consumption rate values remained within a narrow range, 0.185–0.189 g L^−1^ h^−1^ (Table 2). In ADS, succinic acid reached maximum concentrations when fumarate was depleted, as observed in the control assay, but its production rate increased slightly under high alcohol concentration, from 0.106 to 0.134 g L^−1^ h^−1^. Subsequent succinate consumption was also more pronounced than in the control profile (Fig. 1f, i).

In MPW, alcohol exposure produced a different response. The succinic acid production rate decreased from 0.171 g L^−1^ h^−1^ in the control to an average of approximately 0.128 g L^−1^ h^−1^ under alcohol exposure. In contrast, acetic acid accumulation increased relative to the control. The final acetic acid concentration increased from approximately 1.0 g L^−1^ in the control to 1.90 and 2.26 g L^−1^ under low and high alcohol concentrations, respectively. The consortium × alcohol interaction was significant for succinic acid production (*p* < 0.05).

Ethanol and glycerol consumption also differed between consortia. Ethanol consumption showed a highly significant interaction between consortium and alcohol concentration (*p* < 0.01). MPW consumed ethanol at higher rates, averaging approximately 0.017 g L^− 1^ h^− 1^ under alcohol exposure. On the other hand, ADS showed substantially lower rates of 0.004 and 0.005 g L^− 1^ h^− 1^ under the low and high alcohol conditions, respectively (Table 2). Glycerol consumption increased with alcohol concentration in both consortia, but the rates were slightly higher in MPW.

### Carboxylic acids

The effects of carboxylic acids on the two consortia are summarized in Table 3, while the individual duplicate values are provided in Supplementary Table S1. The corresponding profiles are shown in Fig. 1c, g,j for ADS and Fig. 2c, g,j for MPW. In contrast to alcohols, carboxylic acids had a stronger effect on growth and fumarate-linked metabolism. Growth rate was significantly affected by consortium, acid concentration, and the consortium × acid interaction (*p* < 0.001, *p* < 0.001, and *p* = 0.002, respectively; Table 3). MPW decreased from 0.284 h^−1^ in the control to 0.219 h^−1^ at low acid concentration and 0.085 h^−1^ at high acid concentration, corresponding to an approximately 70% reduction. ADS also showed reduced growth, decreasing from 0.181 h^−1^ in the control to 0.113 and 0.106 h^−1^ at low and high acid concentrations, respectively.

Fumarate consumption was also affected by carboxylic acids, particularly at high concentrations. MPW maintained a fumarate consumption rate close to the control at low acid concentration, 0.186 vs. 0.189 g L^−1^ h^−1^, and decreased to 0.162 g L^−1^ h^−1^ at high acid concentration. ADS was more affected, decreasing from 0.186 g L^−1^ h^−1^ in the control to 0.167 and 0.115 g L^−1^ h^−1^ at low and high acid concentrations, respectively. The consortium × acid interaction was significant (*p* < 0.05; Table 3).

The succinic acid production rate showed a highly significant consortium × acid interaction (*p* < 0.001). In ADS, it decreased from 0.106 g L^−1^ h^−1^ in the control to 0.061 and 0.041 g L^−1^ h^−1^ at low and high acid concentrations, respectively. Although the production rate decreased, the maximum succinic acid concentration at high acid concentration exceeded that of the control, reaching 3.57 g L^−1^ after 81 h, compared with 2.61 g L^−1^ after approximately 23 h in the control.

In MPW, the succinic acid production rate remained close to the control at low acid concentration, 0.172 vs. 0.171 g L^−1^ h^−1^, but decreased to 0.074 g L^−1^ h^−1^ at high acid concentration. Acetate dynamics also changed in response to carboxylic acids. In ADS, the acetic acid production rate decreased compared to the control, from 0.028 g L^−1^ h^−1^ to 0.010–0.011 g L^−1^ h^−1^. In MPW, the rate shifted from a negative value in the control, indicating net acetate consumption, to positive values under acid exposure, increasing to 0.013 g L^−1^ h^−1^ at high acid concentration (Table 3). Negative values reflect net consumption over the period considered, since acetate can be simultaneously produced and consumed in anaerobic systems.

The consumption of the added acids differed between compounds and consortia. Lactic acid consumption was detected in both consortia and was higher in ADS than in MPW. Tartaric acid consumption remained comparatively low and variable (Table 3).

### Phenolic compounds

The effects of phenolic compounds are summarized in Table 4, while the individual duplicate values are provided in Supplementary Table S1. The corresponding growth and fermentation profiles are shown in Fig. 1d, h,k for ADS and Fig. 2d, h,k for MPW. Phenolic compounds exerted the clearest inhibitory effect on growth, particularly in the ADS consortium. Both consortium and phenolic concentration significantly affected growth rate, with *p* < 0.001 and *p* < 0.01, respectively (Table 4). MPW decreased from 0.284 h^−1^ in the control to 0.216 and 0.204 h^−1^ at low and high phenolic concentrations, respectively. In contrast, ADS was more sensitive, with the growth rate decreasing from 0.181 h^−1^ in the control to 0.081 and 0.063 h^−1^ under low and high phenolic concentrations, respectively.

The fumarate and succinate data confirmed the difference between the consortia. In ADS, fumarate consumption decreased progressively with increasing phenolic concentration, from 0.186 g L^−1^ h^−1^ in the control to 0.139 and 0.105 g L^−1^ h^−1^ at low and high concentrations, respectively. Succinic acid production also decreased from 0.106 g L^−1^ h^−1^ in the control to approximately 0.055–0.059 g L^−1^ h^−1^ under phenolic exposure (Table 4). By contrast, MPW maintained fumarate consumption and succinic acid production closer to the control profile. Although fumarate consumption rate decreased slightly at low phenolic concentration, MPW retained succinic acid production rates close to the control, with an average of 0.167 g L^−1^ h^−1^ compared with 0.171 g L^−1^ h^−1^ in the control.

Acetic acid production remained low under phenolic exposure. In ADS, acetate production decreased from 0.028 g L^−1^ h^−1^ in the control to 0.017 g L^−1^ h^−1^ at both phenolic concentrations. In MPW, values remained negative or close to zero, indicating low net acetate production (Table 4).

Apparent consumption of the parent phenolic compounds differed between consortia and concentrations. At the high phenolic concentration, catechol consumption rates reached 0.080 g L^− 1^ h^− 1^ in ADS and 0.024 g L^− 1^ h^− 1^ in MPW, compared with 0.002 and 0.007 g L^− 1^ h^− 1^, respectively, at the low concentration. Tyrosol consumption was higher in ADS, reaching 0.153 and 0.081 g L^− 1^ h^− 1^ under the low and high concentration conditions, respectively, compared with 0.019 and 0.033 g L^− 1^ h^− 1^ in MPW (Table 4). These values describe the apparent disappearance of the parent compounds measured by HPLC and should not be interpreted as evidence of complete degradation or mineralization.

### Response to combined inhibitor exposure

**Fig. 3:**
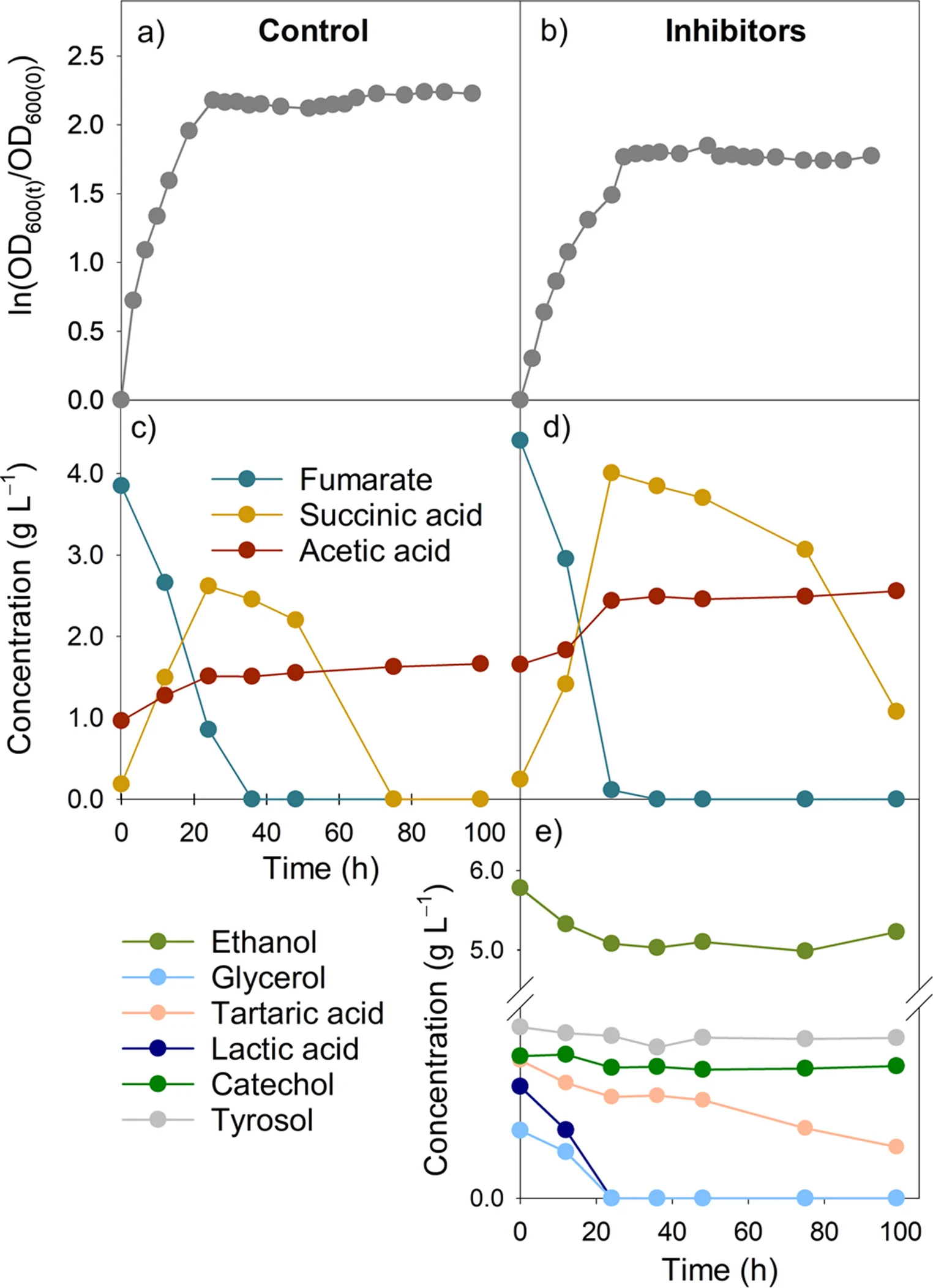
Growth curves and fermentation profiles of the ADS consortium in NBAF medium under control conditions, without inhibitors (**a**, **c**), and in the presence of the combined inhibitor mixture containing alcohols, carboxylic acids, and phenolic compounds at their highest tested concentrations (**b**, **d**, **e**). Error bars were omitted for clarity because replicate values generally differed by less than 10% for the monitored parameters, without affecting the interpretation of the observed trends

**Fig. 4:**
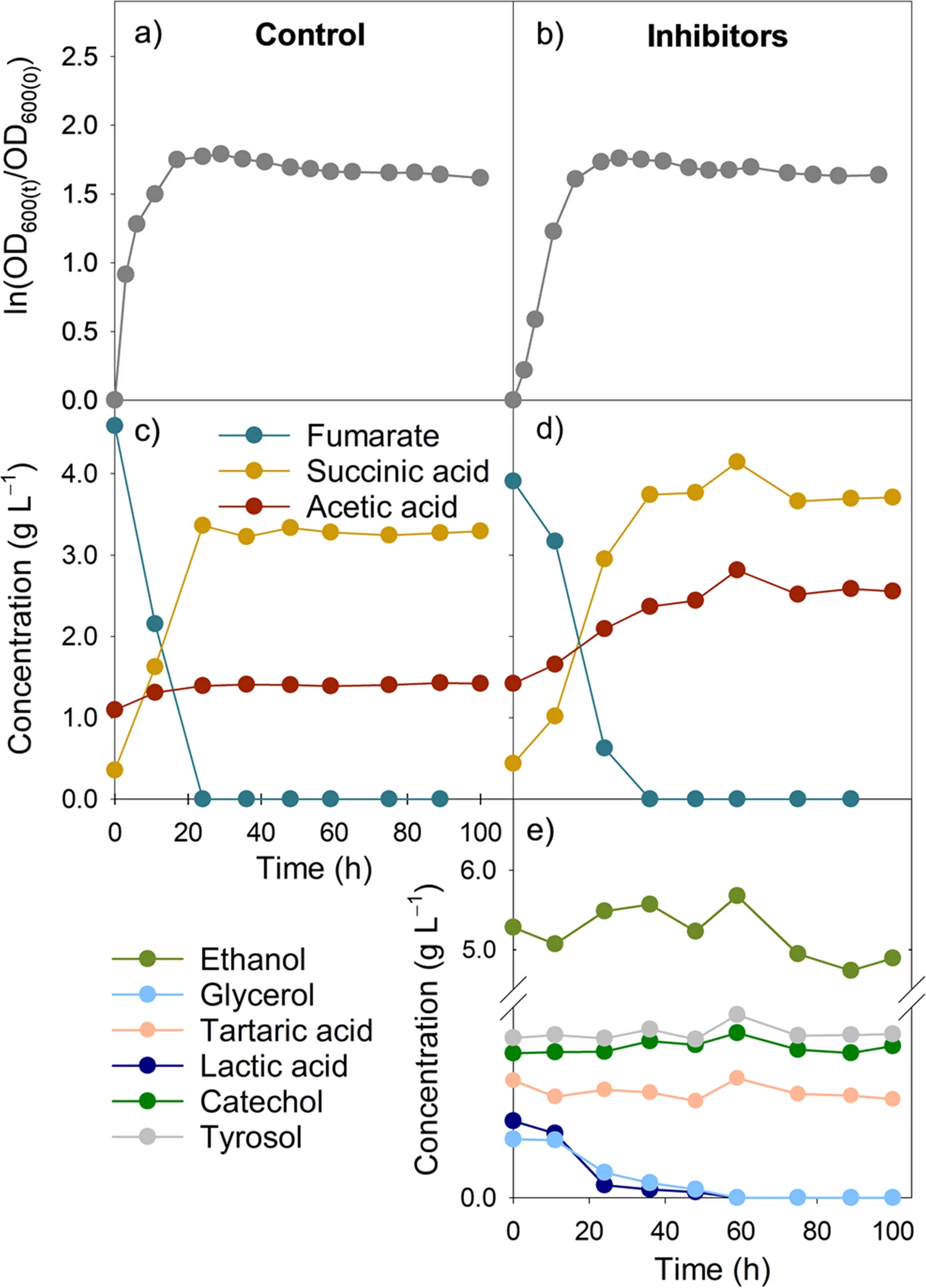
Growth curves and fermentation profiles of the MPW consortium in NBAF medium under control conditions, without inhibitors (**a**, **c**), and in the presence of the combined inhibitor mixture containing alcohols, carboxylic acids, and phenolic compounds at their highest tested concentrations (**b**, **d**, **e**). Error bars were omitted for clarity because replicate values generally differed by less than 10% for the monitored parameters, without affecting the interpretation of the observed trends

The combined inhibitor assays were designed to assess whether the consortia could maintain anaerobic activity when simultaneously exposed to alcohols, carboxylic acids, and phenolic compounds at their highest tested concentrations. The kinetic variables are shown in Table 5, while the individual duplicate values underlying the reported means are provided in Supplementary Table S2. The corresponding growth and fermentation profiles are presented in Fig. 3 for ADS and Fig. 4 for MPW.

**Table 5:** Effects of combined-inhibitor exposure on the growth and metabolic rates of microbial consortia derived from ADS and MPW. The reported p-values correspond to the effects of consortium, combined-inhibitor condition, and the consortium × condition interaction, as determined by two-way analysis of variance. Negative production rates indicate net consumption of the corresponding compound

Variable		Consortium	Combined-inhibitor condition		p-value	
			Control	High concentration		
Growth rate (h^-^^1^)		ADS	0.181	0.106	Consortium Inhibitor Consortium ⨯ Inhibitor	0.006 <0.001 0.003
		MPW	0.305	0.098		
	Fumarate	ADS	0.186	0.179	Consortium Inhibitor Consortium ⨯ Inhibitor	<0.001 <0.001 <0.001
		MPW	0.191	0.137		
Consumption rate (g L^-^^1^ h^-^^1^)	Ethanol	ADS	0.000	0.029	Consortium Inhibitor Consortium ⨯ Inhibitor	0.028 0.012 0.028
		MPW	0.000	0.004		
	Glycerol	ADS	0.000	0.012	Consortium Inhibitor Consortium ⨯ Inhibitor	<0.001 <0.001 <0.001
		MPW	0.000	0.004		
	Tartaric acid	ADS	0.000	0.004	Consortium Inhibitor Consortium ⨯ Inhibitor	<0.001 <0.001 <0.001
		MPW	0.000	0.0002		
	Lactic acid	ADS	0.000	0.020	Consortium Inhibitor Consortium ⨯ Inhibitor	<0.001 <0.001 <0.001
		MPW	0.000	0.006		
	Catechol	ADS	0.000	0.0004	Consortium Inhibitor Consortium ⨯ Inhibitor	0.440 0.007 0.440
		MPW	0.000	0.0003		
	Tyrosol	ADS	0.000	0.002	Consortium Inhibitor Consortium ⨯ Inhibitor	0.495 0.812 0.495
		MPW	0.000	- 0.001		
Production rate (g L^-^^1^ h^-^^1^)	Acetic acid	ADS	0.028	0.033	Consortium Inhibitor Consortium ⨯ Inhibitor	0.024 0.038 0.143
		MPW	0.009	0.026		
	Succinic acid	ADS	0.106	0.157	Consortium Inhibitor Consortium ⨯ Inhibitor	<0.001 <0.001 <0.001
		MPW	0.125	0.092

The combined inhibitor condition had a significant overall effect on growth, and the significant consortium × condition interaction indicated that the magnitude of this response differed between consortia (*p* < 0.001 and *p* = 0.003, respectively; Table 5). MPW decreased from 0.305 h^−1^ in the control to 0.098 h^−1^ under combined inhibitors, while ADS decreased from 0.181 to 0.106 h^−1^ (Table 5). Thus, although MPW had the higher growth rate under control conditions, it showed the larger relative decrease under combined stress.

Fumarate consumption also decreased under combined inhibitors, but the magnitude of the decrease differed between consortia. ADS showed only a small decrease, from 0.186 to 0.179 g L^−1^ h^−1^, whereas MPW decreased from 0.191 to 0.137 g L^−1^ h^−1^ (Table 5). Thus, under simultaneous exposure to alcohols, acids, and phenolics, MPW showed a larger decrease in both growth and fumarate consumption than ADS.

The response of succinic acid production differed markedly between consortia. In ADS, succinic acid production rate increased from 0.106 to 0.157 g L^−1^ h^−1^ under combined inhibitors, but in MPW it decreased from 0.125 to 0.092 g L^−1^ h^−1^ (Table 5). Acetate production increased in both consortia under combined inhibitors, from 0.009 to 0.026 g L^−1^ h^−1^ in MPW and from 0.028 to 0.033 g L^−1^ h^−1^ in ADS (Table 5).

Substrate use patterns under combined exposure differed markedly between consortia. In ADS, the consumption rates of ethanol, tartaric acid, and lactic acid were higher under combined exposure than in the corresponding high-concentration single-class assays. Ethanol consumption increased from 0.005 to 0.029 g L^− 1^ h^− 1^, tartaric acid consumption from 0.001 to 0.004 g L^− 1^ h^− 1^, and lactic acid consumption from 0.018 to 0.020 g L^− 1^ h^− 1^. In contrast, glycerol consumption decreased from 0.022 to 0.012 g L^− 1^ h^− 1^ (Tables 2 and 3, and 5).

In MPW, the consumption rates of ethanol, glycerol, tartaric acid, and lactic acid were all lower under combined exposure than in the corresponding high-concentration single-class assays. Ethanol consumption decreased from 0.019 to 0.004 g L^− 1^ h^− 1^, glycerol consumption from 0.027 to 0.004 g L^− 1^ h^− 1^, tartaric acid consumption from 0.007 to 0.0002 g L^− 1^ h^− 1^, and lactic acid consumption from 0.010 to 0.006 g L^− 1^ h^− 1^ (Tables 2 and 3, and 5). Catechol consumption remained very low in both consortia, with rates of 0.0004 and 0.0003 g L^− 1^ h^− 1^ in ADS and MPW, respectively. Tyrosol showed a low apparent consumption rate in ADS (0.002 g L^− 1^ h^− 1^) and a slightly negative mean value in MPW (− 0.001 g L^− 1^ h^− 1^), indicating no consistent net tyrosol consumption by MPW under the combined condition (Table 5).

### Bacterial composition and diversity of the enriched consortia

**Fig. 5:**
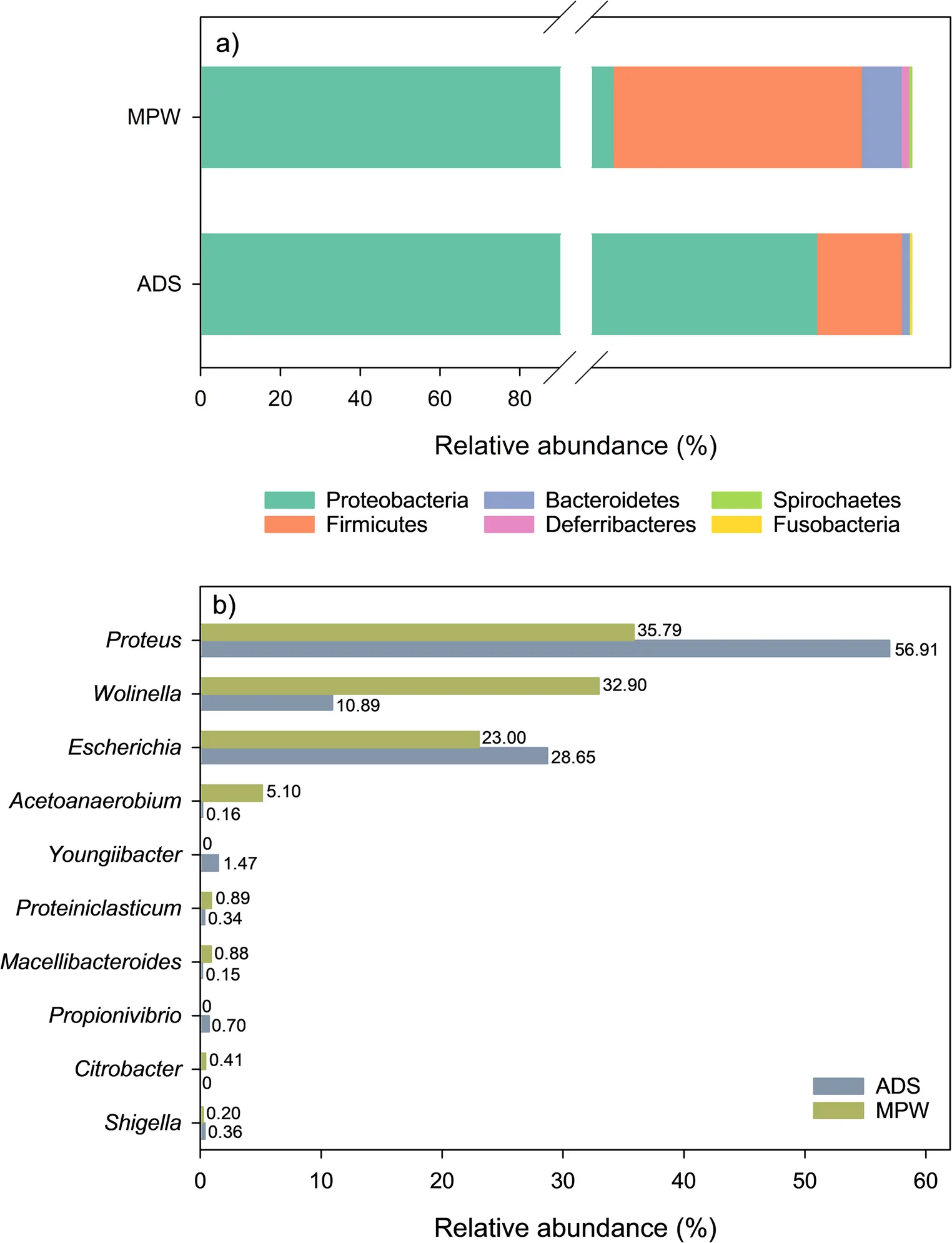
Bacterial taxonomic composition of the enriched ADS and MPW consortia based on full-length 16 S rRNA gene sequencing. Relative abundance presented at the phylum level (**a**) and the ten most abundant genera across both consortia (**b**)

Full-length 16 S rRNA gene sequencing produced datasets containing 90,423 reads for ADS and 111,128 reads for MPW. Only two reads in each dataset remained unassigned, corresponding to taxonomic assignment rates above 99.99%. Both consortia were predominantly composed of Proteobacteria, although their phylum-level distributions differed (Fig. 5a). Proteobacteria represented 97.66% of the ADS consortium and 92.56% of MPW. Firmicutes accounted for 2.12% of ADS and 6.21% of MPW, while Bacteroidetes represented 0.19% and 1.00%, respectively. Deferribacteres were detected only in MPW at 0.19%, while Fusobacteria were detected only in ADS at 0.02%.

More marked differences were observed at the genus level (Fig. 5b). ADS was strongly dominated by *Proteus*, followed by *Escherichia*, *Wolinella*, and *Youngiibacter*. In contrast, MPW showed a more even distribution dominated by *Proteus*, *Wolinella*, and *Escherichia*. *Acetoanaerobium* represented 5.1% of MPW but only 0.16% of ADS. *Proteiniclasticum* and *Macellibacteroides* were also more abundant in MPW. Both consortia contained 20 observed genera. Nevertheless, MPW showed higher genus-level Shannon, Simpson, and Pielou indices than ADS: 1.394 vs. 1.104, 0.707 vs. 0.582, and 0.465 vs. 0.368, respectively (Table 6). The obtained results indicate that the MPW profile was more even and ADS was more strongly dominated by a limited number of genera. The complete taxonomic profiles are provided in Supplementary Table S3.

**Table 6:** Sequencing summary and genus-level bacterial diversity metrics for the enriched ADS and MPW consortia

Parameter	ADS consortium	MPW consortium
Total reads	90,423	111,128
Taxonomically assigned reads (%)	99.998	99.998
Observed genera	20	20
Shannon diversity index	1.104	1.394
Simpson diversity index, 1 − D	0.582	0.707
Pielou evenness index	0.368	0.465

Diversity indices were calculated at the genus level using the estimated taxonomic counts retained after application of the ≥ 0.01% relative-abundance threshold

## Discussion

### Baseline metabolic differences and bacterial composition of the ADS and MPW consortia

The control assays showed that both consortia rapidly consumed fumarate and produced succinate, consistent with fumarate reduction under anaerobic conditions (Schubert and Unden 2023). However, their downstream profiles differed markedly. In ADS, succinate was subsequently consumed while acetate accumulated. In MPW, succinate remained comparatively stable after fumarate depletion and acetate was initially consumed. Since acetate is a central intermediate in anaerobic organic matter degradation, the contrasting profiles indicate differences in the routing of carbon through fermentative, syntrophic, and acetogenic pathways (Pan et al. 2021).

The source environments may have contributed to the initial functional potential of the inocula. Anaerobic digester sludge commonly contains populations involved in organic matter degradation, fermentation, and volatile fatty acid transformation (Pan et al. 2021; Lee et al. 2024). In contrast, the mine wastewater used to obtain MPW originated from a metal and metalloid-affected environment (Dinis et al. 2020). Nevertheless, both consortia were subsequently enriched for three months in the same NBAF medium. Their final bacterial profiles reflect both inoculum source and selective enrichment rather than the original communities directly.

The predominance of *Proteus*, *Escherichia*, and *Wolinella* in both consortia aligns with their strong fumarate-reducing capacity, as representatives of these genera can perform fumarate-linked anaerobic metabolism (Van der Beek et al. 1976; Kröger et al. 2002; Schubert and Unden 2023). The higher relative abundance of *Wolinella* in MPW aligns with the higher baseline succinate production rate, as *W. succinogenes* is specialized in fumarate respiration (Kröger et al. 2002). ADS, which was more strongly dominated by *Proteus* and *Escherichia*, showed greater succinate turnover and net acetate accumulation. By contrast, the greater abundance of *Acetoanaerobium* and the more even bacterial composition of MPW are compatible with a wider contribution from anaerobic fermentative populations (Fonknechten et al. 2010). The 16 S rRNA data help to interpret the metabolic differences, but they do not identify which taxa were active during the assays.

### Effect of alcohols on anaerobic carbon flow

Alcohols caused little inhibition of fumarate consumption and appear to have acted mainly as additional fermentable substrates. In ADS, the increase in succinate production rate at the high alcohol concentration, followed by subsequent succinate consumption, suggests redistribution of carbon and reducing equivalents between fumarate-linked and acetate-associated pathways. Similar relationships between anaerobic organic matter degradation and acetate-centered metabolism have been reported previously (Pan et al. 2021). Succinate and acetate production under anaerobic conditions has also been described in enteric bacteria, including *E. coli* (Schubert and Unden 2023), one of the dominant taxa in ADS.

MPW consumed ethanol and glycerol more rapidly and shifted from net acetate consumption under control conditions to acetate production in the presence of alcohols. The temporal association between alcohol consumption and acetate accumulation suggests that part of the additional carbon was directed towards acetate formation. This broader alcohol utilization response is in line with the greater bacterial evenness of MPW and its higher contribution of anaerobic fermentative taxa. Thus, the response to alcohols was influenced not only by concentration but also by the source and enrichment history of the inocula and the resulting bacterial composition.

### Effect of carboxylic acids on growth and fumarate-linked metabolism

Carboxylic acids exerted stronger effects than alcohols on both growth and fumarate-linked metabolism. MPW largely maintained fumarate consumption under the low acid condition, although ADS showed a clearer decrease. At the highest concentration, both consortia were impaired. The higher abundance of *Wolinella* in MPW is in accordance with its strong fumarate-reducing potential (Kröger et al. 2002), but this taxonomic potential did not prevent reductions in growth and succinate production under stronger acid stress. In ADS, the lower succinate production rate reflected delayed rather than completely suppressed conversion, since a comparatively high maximum succinate concentration was reached after prolonged incubation. Thus, the endpoint concentration revealed a delayed conversion that was not evident from the production rate alone.

The effects of the acid mixtures probably resulted from both substrate-specific effects and the associated decrease in initial pH from 7.04 in the control to 6.60 and 6.02 under the low- and high-acid conditions, respectively (Table 1). Organic acids may serve as metabolic substrates or intermediates, but at elevated concentrations they can affect intracellular pH, transport, and redox balance, particularly through an increased fraction of undissociated acid (Schubert and Unden 2022; Parshina et al. 2023; Gaspar et al. 2025). The different consumption patterns of lactic and tartaric acids further show that acid exposure altered both fumarate-linked activity and carbon source utilization.

### Effect of phenolic compounds on anaerobic activity

Phenolic compounds were the most selective stressors. ADS showed pronounced reductions in growth, fumarate consumption, and succinate production. MPW maintained fumarate-linked metabolism closer to its control profile. This contrast occurred despite the lower apparent catechol and tyrosol consumption rates observed in MPW, indicating that tolerance to phenolic exposure was not directly dependent on extensive removal of the parent compounds. The inhibitory effects observed are consistent with previous reports on anaerobic systems. Hernández and Edyvean (2008) showed that phenolic compounds, including catechol and tyrosol, inhibited biogas production and biodegradability in sludge-based anaerobic processes, with the magnitude of inhibition increasing with concentration. Similarly, Alves-Ferreira et al. (2022) reported that removing phenolic compounds from *Cistus ladanifer* hydrolysates improved D-lactic acid production by *Escherichia coli* JU15 and reduced the time required to reach the maximum product concentration. Together with the present results, these studies indicate that phenolics can slow anaerobic carbon conversion without completely stopping microbial activity.

Despite its stronger metabolic inhibition, ADS showed greater apparent consumption of catechol and tyrosol than MPW, particularly for tyrosol at the low concentration. Thus, disappearance of the parent compounds was not directly associated with preservation of growth or fumarate-linked activity. Moreover, HPLC quantified only changes in the parent compounds and did not demonstrate complete biodegradation or mineralization. Apparent consumption may have involved adsorption, uptake, or partial transformation into unidentified intermediates.

Anaerobic transformation of phenolic compounds requires specialized pathways for aromatic ring activation and cleavage (Schink et al. 2000; Levén et al. 2012; Tomei et al. 2021). The compound- and concentration-dependent disappearance observed in the individual phenolic assays suggests that some transformation occurred, particularly in ADS. However, catechol and tyrosol consumption became minimal when they were present in the combined inhibitor mixture, indicating that the simultaneous availability of other substrates and inhibitors strongly limited phenolic transformation.

The greater bacterial evenness of MPW may have contributed to the maintenance of fumarate-linked activity under phenolic exposure by providing a distribution of metabolic functions and reducing dependence on a small number of dominant populations. This indicates that phenolic tolerance and phenolic transformation should be evaluated independently when selecting consortia for wastewaters containing phenolics.

### Response to combined inhibitor exposure

The combined medium reproduced, under controlled conditions, the simultaneous presence of alcohols, organic acids, and phenolics in winery wastewater. Both consortia retained anaerobic activity, but their responses differed from those observed in the single-class assays.

MPW showed the larger decreases in growth and fumarate consumption despite its higher baseline activity and greater bacterial evenness. In contrast, ADS largely maintained fumarate consumption. Succinate production also responded oppositely: it increased in ADS but decreased in MPW. The ADS response may reflect greater availability of fermentable substrates and redistribution of reducing equivalents towards fumarate-linked metabolism. On the other hand, the simultaneous decreases in fumarate consumption and succinate production in MPW indicate a larger limitation of this pathway.

Substrate selection also differed. In ADS, combined exposure enhanced ethanol, tartaric acid, and lactic acid consumption relative to the corresponding high-concentration single-class assays, while glycerol consumption decreased. MPW showed lower consumption of all four substrates under combined exposure. Catechol and tyrosol consumption remained minimal, showing that the higher substrate use observed in ADS did not extend to phenolic transformation.

Acetate production increased in both consortia, confirming that the mixture altered the distribution of carbon and reducing equivalents rather than simply suppressing activity. The maintenance of measurable conversion under simultaneous exposure is pertinent to real winery wastewater, where microbial communities must tolerate fluctuations in both inhibitor load and substrate availability. Such behavior is in line with functional persistence in complex biological systems (Ngwenya et al. 2022; Flores et al. 2023) and with fermentation responses previously observed in inhibitor-containing hydrolysates (Alves-Ferreira et al. 2022). The results also show that responses to combined inhibitors cannot be inferred directly from single-class assays.

### Integrated interpretation and implications

The data from taxonomic and metabolic analysis showed that the performance of the consortia depended on the conditions. MPW combined higher baseline activity, stronger alcohol utilization, and greater maintenance of fumarate-linked metabolism under moderate acid and phenolic exposure. ADS showed greater sensitivity to phenolics but maintained fumarate consumption and increased succinate production under combined exposure. Therefore, neither bacterial diversity nor growth alone was sufficient to predict functional resilience. Fumarate consumption, succinate formation, acetate turnover, and individual compound utilization provided complementary indicators of metabolic performance.

The results were interpreted by considering both the statistical evidence and the magnitude and consistency of the responses across the measured variables. Each condition was tested in two independently prepared flasks, and taxonomic profiling was performed using one sequencing library per consortium. Thus, the ANOVA results and diversity indices were used as supporting, rather than conclusive, evidence. Full-length 16 S rRNA sequencing described the bacterial composition of each consortium but could not identify active populations, gene expression, or functional genes. Archaeal and fungal communities were outside the scope of the present study.

The advantages presented by ADS and MPW indicate that consortium selection should take the expected wastewater composition into account, rather than rely on a single performance indicator. Fumarate consumption, succinate formation, acetate turnover, and compound utilization should be considered together when selecting consortia for biological and bioelectrochemical applications. Where phenolic transformation is a priority, targeted acclimation or enrichment may also be required.

## Conclusion

This study showed that microbial consortia derived from anaerobic digester sludge and mining process wastewater, and subsequently enriched under the same conditions, developed distinct bacterial compositions and anaerobic metabolic profiles. Both consortia maintained fumarate-linked activity, but ADS showed greater succinate turnover and net acetate production. At the same time, MPW exhibited a higher baseline succinic acid production rate and stronger ethanol and glycerol utilization. Such differences were consistent with their bacterial profiles: ADS was mainly dominated by *Proteus* and *Escherichia*, while MPW showed a more even community with greater relative contributions from *Wolinella* and *Acetoanaerobium*.

The response varied with both consortium and compound class. Alcohols acted mainly as additional fermentable substrates, carboxylic acids delayed fumarate-linked conversion, and phenolic compounds were the most selective stressors. MPW maintained fumarate reduction more effectively under phenolic exposure, but ADS showed greater apparent removal of catechol and tyrosol but stronger metabolic inhibition, demonstrating that phenolic tolerance and phenolic transformation are distinct properties. Under combined exposure, ADS maintained fumarate consumption and increased succinic acid production, while MPW showed visible reductions in substrate utilization and fumarate-linked metabolism.

Consortium performance cannot be predicted from growth, bacterial diversity, or environmental origin alone. Combining metabolic indicators with bacterial composition provides a better basis for selecting consortia according to the expected wastewater composition. This can support the development of more resilient biological and bioelectrochemical processes for the biodegradation and sustainable valorization of complex agro-industrial wastewaters. Targeted acclimation or enrichment may be particularly relevant for improving phenolic transformation.

## Supplementary Information

Below is the link to the electronic supplementary material.

Supplementary Material 1

## Data Availability

Data and materials will be made available on request.
