# Supplementary material for: Anaerobic metabolic responses of sludge and mine wastewater-derived consortia to winery wastewater-related compounds
Source: World J Microbiol Biotechnol. 2026 Aug 18;42(9):475. doi: 10.1007/s11274-026-05195-3 (PMC13486130; doi:10.1007/s11274-026-05195-3)
Supplement: Supplementary file 1 [file 11274_2026_5195_MOESM1_ESM.docx]

**Anaerobic metabolic responses of sludge and mine wastewater-derived consortia to winery wastewater-related compounds**

Ana Baía^1,2^, Alonso I. Arroyo-Escoto^3,4,5^, Nuno Ramos^2^, César Afonso^1,2^, Gaetano R. Pesce^6^, Maria C. Fernandes^3,4,*^, Annabel Fernandes^1,2,*^

**Supplementary Material**

**Table S1.** Individual duplicate values underlying the mean growth and metabolic rates reported in Tables 2–4 for the ADS and MPW consortia exposed to alcohols, carboxylic acids, and phenolic compounds. Zero values indicate that the corresponding compound was not present or that no net consumption or production was detected

| Growth rate (h^−1^) | | | | | | | |
| --- | --- | --- | --- | --- | --- | --- | --- |
| Consortium | Inhibitor concentration | Inhibitors | | | | | |
|  |  | Alcohol | | Carboxylic acid | | Phenolic compound | |
| ADS | Control | 0.200 | 0.163 | 0.200 | 0.163 | 0.200 | 0.163 |
| ADS | Low | 0.208 | 0.226 | 0.111 | 0.116 | 0.083 | 0.078 |
| ADS | High | 0.195 | 0.184 | 0.107 | 0.104 | 0.070 | 0.055 |
| MPW | Control | 0.281 | 0.288 | 0.281 | 0.288 | 0.281 | 0.288 |
| MPW | Low | 0.311 | 0.293 | 0.238 | 0.200 | 0.193 | 0.239 |
| MPW | High | 0.334 | 0.309 | 0.087 | 0.083 | 0.224 | 0.185 |
| Fumarate consumption rate (g L^−1^ h^−1^) | | | | | | | |
| Consortium | Inhibitor concentration | Inhibitors | | | | | |
|  |  | Alcohol | | Carboxylic acid | | Phenolic compound | |
| ADS | Control | 0.186 | 0.186 | 0.186 | 0.186 | 0.186 | 0.186 |
| ADS | Low | 0.188 | 0.188 | 0.160 | 0.174 | 0.140 | 0.139 |
| ADS | High | 0.186 | 0.184 | 0.106 | 0.124 | 0.102 | 0.108 |
| MPW | Control | 0.189 | 0.190 | 0.189 | 0.190 | 0.189 | 0.190 |
| MPW | Low | 0.183 | 0.187 | 0.187 | 0.186 | 0.166 | 0.180 |
| MPW | High | 0.186 | 0.184 | 0.153 | 0.170 | 0.179 | 0.187 |
| Ethanol consumption rate (g L^−1^ h^−1^) | | | | | | | |
| Consortium | Inhibitor concentration | Inhibitors | | | | | |
|  |  | Alcohol | | Carboxylic acid | | Phenolic compound | |
| ADS | Control | 0.000 | 0.000 | 0.000 | 0.000 | 0.000 | 0.000 |
| ADS | Low | 0.002 | 0.006 | 0.000 | 0.000 | 0.000 | 0.000 |
| ADS | High | 0.002 | 0.007 | 0.000 | 0.000 | 0.000 | 0.000 |
| MPW | Control | 0.000 | 0.000 | 0.000 | 0.000 | 0.000 | 0.000 |
| MPW | Low | 0.016 | 0.015 | 0.000 | 0.000 | 0.000 | 0.000 |
| MPW | High | 0.021 | 0.018 | 0.000 | 0.000 | 0.000 | 0.000 |
| Glycerol consumption rate (g L^−1^ h^−1^) | | | | | | | |
| Consortium | Inhibitor concentration | Inhibitors | | | | | |
|  |  | Alcohol | | Carboxylic acid | | Phenolic compound | |
| ADS | Control | 0.000 | 0.000 | 0.000 | 0.000 | 0.000 | 0.000 |
| ADS | Low | 0.012 | 0.012 | 0.000 | 0.000 | 0.000 | 0.000 |
| ADS | High | 0.023 | 0.021 | 0.000 | 0.000 | 0.000 | 0.000 |
| MPW | Control | 0.000 | 0.000 | 0.000 | 0.000 | 0.000 | 0.000 |
| MPW | Low | 0.014 | 0.014 | 0.000 | 0.000 | 0.000 | 0.000 |
| MPW | High | 0.027 | 0.027 | 0.000 | 0.000 | 0.000 | 0.000 |
| Tartaric acid consumption rate (g L^−1^ h^−1^) | | | | | | | |
| Consortium | Inhibitor concentration | Inhibitors | | | | | |
|  |  | Alcohol | | Carboxylic acid | | Phenolic compound | |
| ADS | Control | 0.000 | 0.000 | 0.000 | 0.000 | 0.000 | 0.000 |
| ADS | Low | 0.000 | 0.000 | 0.006 | 0.006 | 0.000 | 0.000 |
| ADS | High | 0.000 | 0.000 | 0.001 | 0.001 | 0.000 | 0.000 |
| MPW | Control | 0.000 | 0.000 | 0.000 | 0.000 | 0.000 | 0.000 |
| MPW | Low | 0.000 | 0.000 | 0.005 | 0.005 | 0.000 | 0.000 |
| MPW | High | 0.000 | 0.000 | 0.007 | 0.006 | 0.000 | 0.000 |
| Lactic acid consumption rate (g L^−1^ h^−1^) | | | | | | | |
| Consortium | Inhibitor concentration | Inhibitors | | | | | |
|  |  | Alcohol | | Carboxylic acid | | Phenolic compound | |
| ADS | Control | 0.000 | 0.000 | 0.000 | 0.000 | 0.000 | 0.000 |
| ADS | Low | 0.000 | 0.000 | 0.021 | 0.023 | 0.000 | 0.000 |
| ADS | High | 0.000 | 0.000 | 0.018 | 0.018 | 0.000 | 0.000 |
| MPW | Control | 0.000 | 0.000 | 0.000 | 0.000 | 0.000 | 0.000 |
| MPW | Low | 0.000 | 0.000 | 0.010 | 0.010 | 0.000 | 0.000 |
| MPW | High | 0.000 | 0.000 | 0.010 | 0.010 | 0.000 | 0.000 |
| Catechol consumption rate (g L^−1^ h^−1^) | | | | | | | |
| Consortium | Inhibitor concentration | Inhibitors | | | | | |
|  |  | Alcohol | | Carboxylic acid | | Phenolic compound | |
| ADS | Control | 0.000 | 0.000 | 0.000 | 0.000 | 0.000 | 0.000 |
| ADS | Low | 0.000 | 0.000 | 0.000 | 0.000 | 0.003 | 0.000 |
| ADS | High | 0.000 | 0.000 | 0.000 | 0.000 | 0.088 | 0.071 |
| MPW | Control | 0.000 | 0.000 | 0.000 | 0.000 | 0.000 | 0.000 |
| MPW | Low | 0.000 | 0.000 | 0.000 | 0.000 | 0.001 | 0.012 |
| MPW | High | 0.000 | 0.000 | 0.000 | 0.000 | 0.016 | 0.032 |
| Tyrosol consumption rate (g L^−1^ h^−1^) | | | | | | | |
| Consortium | Inhibitor concentration | Inhibitors | | | | | |
|  |  | Alcohol | | Carboxylic acid | | Phenolic compound | |
| ADS | Control | 0.000 | 0.000 | 0.000 | 0.000 | 0.000 | 0.000 |
| ADS | Low | 0.000 | 0.000 | 0.000 | 0.000 | 0.153 | 0.153 |
| ADS | High | 0.000 | 0.000 | 0.000 | 0.000 | 0.090 | 0.073 |
| MPW | Control | 0.000 | 0.000 | 0.000 | 0.000 | 0.000 | 0.000 |
| MPW | Low | 0.000 | 0.000 | 0.000 | 0.000 | 0.017 | 0.021 |
| MPW | High | 0.000 | 0.000 | 0.000 | 0.000 | 0.031 | 0.036 |
| Acetic acid production rate (g L^−1^ h^−1^) | | | | | | | |
| Consortium | Inhibitor concentration | Inhibitors | | | | | |
|  |  | Alcohol | | Carboxylic acid | | Phenolic compound | |
| ADS | Control | 0.035 | 0.021 | 0.035 | 0.021 | 0.035 | 0.021 |
| ADS | Low | 0.046 | 0.019 | 0.009 | 0.011 | 0.018 | 0.016 |
| ADS | High | 0.047 | 0.032 | 0.012 | 0.010 | 0.017 | 0.016 |
| MPW | Control | −0.010 | −0.011 | −0.010 | −0.011 | −0.010 | −0.011 |
| MPW | Low | 0.007 | 0.005 | 0.001 | 0.001 | −0.008 | −0.005 |
| MPW | High | 0.011 | 0.011 | 0.016 | 0.011 | 0.000 | −0.004 |
| Succinic acid production rate (g L^−1^ h^−1^) | | | | | | | |
| Consortium | Inhibitor concentration | Inhibitors | | | | | |
|  |  | Alcohol | | Carboxylic acid | | Phenolic compound | |
| ADS | Control | 0.106 | 0.105 | 0.106 | 0.105 | 0.106 | 0.105 |
| ADS | Low | 0.137 | 0.097 | 0.062 | 0.060 | 0.056 | 0.055 |
| ADS | High | 0.145 | 0.123 | 0.041 | 0.040 | 0.058 | 0.060 |
| MPW | Control | 0.169 | 0.172 | 0.169 | 0.172 | 0.169 | 0.172 |
| MPW | Low | 0.124 | 0.123 | 0.172 | 0.172 | 0.169 | 0.162 |
| MPW | High | 0.136 | 0.131 | 0.074 | 0.073 | 0.166 | 0.171 |

**Table S2.** Individual duplicate values underlying the mean growth and metabolic rates reported in Table 5 for the ADS and MPW consortia under the combined-inhibitor condition. Zero values indicate that no net consumption or production was detected. Negative values indicate net production rather than consumption, or net consumption rather than production, according to the variable reported

| Consortium | Inhibitor concentration | Growth rate (h^−1^) | |
| --- | --- | --- | --- |
| ADS | Control | 0.200 | 0.163 |
| ADS | High | 0.099 | 0.114 |
| MPW | Control | 0.303 | 0.306 |
| MPW | High | 0.091 | 0.105 |
| Consortium | Inhibitor concentration | Fumarate consumption rate (g L^−1^ h^−1^) | |
| ADS | Control | 0.186 | 0.186 |
| ADS | High | 0.181 | 0.177 |
| MPW | Control | 0.194 | 0.188 |
| MPW | High | 0.137 | 0.137 |
| Consortium | Inhibitor concentration | Ethanol consumption rate (g L^−1^ h^−1^) | |
| ADS | Control | 0.000 | 0.000 |
| ADS | High | 0.022 | 0.037 |
| MPW | Control | 0.000 | 0.000 |
| MPW | High | 0.003 | 0.005 |
| Consortium | Inhibitor concentration | Glycerol consumption rate (g L^−1^ h^−1^) | |
| ADS | Control | 0.000 | 0.000 |
| ADS | High | 0.013 | 0.011 |
| MPW | Control | 0.000 | 0.000 |
| MPW | High | 0.004 | 0.004 |
| Consortium | Inhibitor concentration | Tartaric acid consumption rate (g L^−1^ h^−1^) | |
| ADS | Control | 0.000 | 0.000 |
| ADS | High | 0.004 | 0.004 |
| MPW | Control | 0.000 | 0.000 |
| MPW | High | 0.0001 | 0.0003 |
| Consortium | Inhibitor concentration | Lactic acid consumption rate (g L^−1^ h^−1^) | |
| ADS | Control | 0.000 | 0.000 |
| ADS | High | 0.020 | 0.019 |
| MPW | Control | 0.000 | 0.000 |
| MPW | High | 0.006 | 0.006 |
| Consortium | Inhibitor concentration | Catechol consumption rate (g L^−1^ h^−1^) | |
| ADS | Control | 0.0000 | 0.0000 |
| ADS | High | 0.0003 | 0.0006 |
| MPW | Control | 0.0000 | 0.0000 |
| MPW | High | 0.0003 | 0.0003 |
| Consortium | Inhibitor concentration | Tyrosol consumption rate (g L^−1^ h^−1^) | |
| ADS | Control | 0.000 | 0.000 |
| ADS | High | 0.002 | 0.002 |
| MPW | Control | 0.000 | 0.000 |
| MPW | High | 0.003 | −0.005 |
| Consortium | Inhibitor concentration | Acetic acid production rate (g L^−1^ h^−1^) | |
| ADS | Control | 0.035 | 0.021 |
| ADS | High | 0.032 | 0.033 |
| MPW | Control | 0.007 | 0.010 |
| MPW | High | 0.027 | 0.026 |
| Consortium | Inhibitor concentration | Succinic acid production rate (g L^−1^ h^−1^) | |
| ADS | Control | 0.106 | 0.105 |
| ADS | High | 0.156 | 0.158 |
| MPW | Control | 0.125 | 0.126 |
| MPW | High | 0.091 | 0.092 |

**Table S3.** Bacterial taxonomic profile of the enriched ADS and MPW consortia obtained by full-length 16S rRNA gene sequencing

| Species-level assignment | Genus | Family | Order | Class | Phylum | ADS relative abundance (%) | MPW relative abundance (%) |
| --- | --- | --- | --- | --- | --- | --- | --- |
| *Proteus vulgaris* | *Proteus* | Morganellaceae | Enterobacterales | Gammaproteobacteria | Proteobacteria | 56.426 | 32.795 |
| *Wolinella succinogenes* | *Wolinella* | Helicobacteraceae | Campylobacterales | Epsilonproteobacteria | Proteobacteria | 10.891 | 32.905 |
| *Escherichia coli* | *Escherichia* | Enterobacteriaceae | Enterobacterales | Gammaproteobacteria | Proteobacteria | 28.648 | 22.962 |
| *Acetoanaerobium sticklandii* | *Acetoanaerobium* | Peptostreptococcaceae | Clostridiales | Clostridia | Firmicutes | 0.162 | 5.036 |
| *Proteus cibarius* | *Proteus* | Morganellaceae | Enterobacterales | Gammaproteobacteria | Proteobacteria | — | 1.980 |
| *Youngiibacter multivorans* | *Youngiibacter* | Clostridiaceae | Clostridiales | Clostridia | Firmicutes | 1.378 | — |
| *Proteiniclasticum ruminis* | *Proteiniclasticum* | Clostridiaceae | Clostridiales | Clostridia | Firmicutes | 0.339 | 0.888 |
| *Macellibacteroides fermentans* | *Macellibacteroides* | Porphyromonadaceae | Bacteroidales | Bacteroidia | Bacteroidetes | 0.155 | 0.877 |
| *Propionivibrio pelophilus* | *Propionivibrio* | Rhodocyclaceae | Rhodocyclales | Betaproteobacteria | Proteobacteria | 0.696 | — |
| *Proteus hauseri* | *Proteus* | Morganellaceae | Enterobacterales | Gammaproteobacteria | Proteobacteria | 0.033 | 0.476 |
| *Citrobacter freundii* | *Citrobacter* | Enterobacteriaceae | Enterobacterales | Gammaproteobacteria | Proteobacteria | — | 0.392 |
| *Proteus penneri* | *Proteus* | Morganellaceae | Enterobacterales | Gammaproteobacteria | Proteobacteria | 0.108 | 0.250 |
| *Shigella boydii* | *Shigella* | Enterobacteriaceae | Enterobacterales | Gammaproteobacteria | Proteobacteria | 0.245 | 0.022 |
| *Proteus alimentorum* | *Proteus* | Morganellaceae | Enterobacterales | Gammaproteobacteria | Proteobacteria | 0.230 | 0.009 |
| *Geovibrio thiophilus* | *Geovibrio* | Deferribacteraceae | Deferribacterales | Deferribacteres | Deferribacteres | — | 0.190 |
| *Proteus terrae* | *Proteus* | Morganellaceae | Enterobacterales | Gammaproteobacteria | Proteobacteria | — | 0.165 |
| *Laceyella sacchari* | *Laceyella* | Thermoactinomycetaceae | Bacillales | Bacilli | Firmicutes | 0.044 | 0.133 |
| *Shigella flexneri* | *Shigella* | Enterobacteriaceae | Enterobacterales | Gammaproteobacteria | Proteobacteria | 0.085 | 0.125 |
| *Enterobacter cloacae* | *Enterobacter* | Enterobacteriaceae | Enterobacterales | Gammaproteobacteria | Proteobacteria | 0.073 | 0.097 |
| *Youngiibacter fragilis* | *Youngiibacter* | Clostridiaceae | Clostridiales | Clostridia | Firmicutes | 0.089 | — |
| *Proteus mirabilis* | *Proteus* | Morganellaceae | Enterobacterales | Gammaproteobacteria | Proteobacteria | 0.043 | 0.088 |
| *Proteus sp. CD3* | *Proteus* | Morganellaceae | Enterobacterales | Gammaproteobacteria | Proteobacteria | 0.068 | 0.017 |
| *Acetoanaerobium noterae* | *Acetoanaerobium* | Peptostreptococcaceae | Clostridiales | Clostridia | Firmicutes | — | 0.063 |
| *Parabacteroides chartae* | *Parabacteroides* | Tannerellaceae | Bacteroidales | Bacteroidia | Bacteroidetes | 0.013 | 0.055 |
| *Enterococcus gallinarum* | *Enterococcus* | Enterococcaceae | Lactobacillales | Bacilli | Firmicutes | 0.046 | 0.032 |
| Bacteroidales bacterium CF | Unclassified | Unclassified | Bacteroidales | Bacteroidia | Bacteroidetes | — | 0.041 |
| Enterobacteriaceae bacterium strain FGI 57 | Unclassified | Enterobacteriaceae | Enterobacterales | Gammaproteobacteria | Proteobacteria | 0.033 | 0.041 |
| *Enterocloster aldensis* | *Enterocloster* | Lachnospiraceae | Clostridiales | Clostridia | Firmicutes | 0.039 | — |
| *Enterobacter hormaechei* | *Enterobacter* | Enterobacteriaceae | Enterobacterales | Gammaproteobacteria | Proteobacteria | — | 0.038 |
| *Shigella sonnei* | *Shigella* | Enterobacteriaceae | Enterobacterales | Gammaproteobacteria | Proteobacteria | 0.028 | 0.032 |
| *Enterococcus sp. M190262* | *Enterococcus* | Enterococcaceae | Lactobacillales | Bacilli | Firmicutes | — | 0.031 |
| *Enterococcus sp. FDAARGOS_553* | *Enterococcus* | Enterococcaceae | Lactobacillales | Bacilli | Firmicutes | — | 0.029 |
| *Sphaerochaeta associata* | *Sphaerochaeta* | Spirochaetaceae | Spirochaetales | Spirochaetia | Spirochaetes | 0.012 | 0.026 |
| *Escherichia fergusonii* | *Escherichia* | Enterobacteriaceae | Enterobacterales | Gammaproteobacteria | Proteobacteria | — | 0.024 |
| *Enterococcus avium* | *Enterococcus* | Enterococcaceae | Lactobacillales | Bacilli | Firmicutes | 0.023 | — |
| *Salmonella enterica* | *Salmonella* | Enterobacteriaceae | Enterobacterales | Gammaproteobacteria | Proteobacteria | 0.016 | 0.022 |
| *Shigella dysenteriae* | *Shigella* | Enterobacteriaceae | Enterobacterales | Gammaproteobacteria | Proteobacteria | — | 0.022 |
| *Mixta calida* | *Mixta* | Erwiniaceae | Enterobacterales | Gammaproteobacteria | Proteobacteria | — | 0.022 |
| *Providencia rettgeri* | *Providencia* | Morganellaceae | Enterobacterales | Gammaproteobacteria | Proteobacteria | — | 0.020 |
| *Citrobacter sp. FDAARGOS_156* | *Citrobacter* | Enterobacteriaceae | Enterobacterales | Gammaproteobacteria | Proteobacteria | — | 0.020 |
| *Desulfovibrio desulfuricans* | *Desulfovibrio* | Desulfovibrionaceae | Desulfovibrionales | Deltaproteobacteria | Proteobacteria | 0.019 | — |
| *Fusobacterium ulcerans* | *Fusobacterium* | Fusobacteriaceae | Fusobacteriales | Fusobacteriia | Fusobacteria | 0.015 | — |
| *Edwardsiella sp. EA181011* | *Edwardsiella* | Hafniaceae | Enterobacterales | Gammaproteobacteria | Proteobacteria | — | 0.015 |
| *Xenorhabdus hominickii* | *Xenorhabdus* | Morganellaceae | Enterobacterales | Gammaproteobacteria | Proteobacteria | 0.015 | — |
| *Lentimicrobium saccharophilum* | *Lentimicrobium* | Lentimicrobiaceae | Bacteroidales | Bacteroidia | Bacteroidetes | — | 0.014 |
| *Bacteroides graminisolvens* | *Bacteroides* | Bacteroidaceae | Bacteroidales | Bacteroidia | Bacteroidetes | 0.013 | — |
| *Escherichia albertii* | *Escherichia* | Enterobacteriaceae | Enterobacterales | Gammaproteobacteria | Proteobacteria | — | 0.012 |
| *Bacteroides thetaiotaomicron* | *Bacteroides* | Bacteroidaceae | Bacteroidales | Bacteroidia | Bacteroidetes | 0.011 | — |
| *Proteus columbae* | *Proteus* | Morganellaceae | Enterobacterales | Gammaproteobacteria | Proteobacteria | — | 0.011 |
| *Sphaerochaeta globosa* | *Sphaerochaeta* | Spirochaetaceae | Spirochaetales | Spirochaetia | Spirochaetes | — | 0.010 |
| *Dysgonomonas mossii* | *Dysgonomonas* | Dysgonomonadaceae | Bacteroidales | Bacteroidia | Bacteroidetes | — | 0.010 |

Relative abundances were calculated from the estimated taxonomic counts produced by the wf-emu workflow. Taxa retained at a relative abundance of ≥0.01% in at least one consortium are presented; —, not detected or not retained above the reporting threshold. Species-level assignments derived from full-length 16S rRNA gene data should be interpreted cautiously, particularly for closely related taxa. Values represent one sequencing library per enriched consortium.
